# A Simple BODIPY-Based Viscosity Probe for Imaging of Cellular Viscosity in Live Cells

**DOI:** 10.3390/s16091397

**Published:** 2016-08-31

**Authors:** Dongdong Su, Chai Lean Teoh, Nengyue Gao, Qing-Hua Xu, Young-Tae Chang

**Affiliations:** 1 Singapore Bioimaging Consortium, Agency for Science, Technology and Research (A*STAR), Singapore 138667, Singapore; sudongdong1984@gmail.com (D.S.); chai.teoh@gmail.com (C.L.T.); 2 Department of Chemistry, National University of Singapore, Singapore 117543, Singapore; chmgn@nus.edu.sg (N.G.); chmxqh@nus.edu.sg (Q.-H.X.)

**Keywords:** BODIPY, fluorescent probes, sensors, viscosity, FLIM, cell imaging

## Abstract

Intracellular viscosity is a fundamental physical parameter that indicates the functioning of cells. In this work, we developed a simple boron-dipyrromethene (BODIPY)-based probe, **BTV**, for cellular mitochondria viscosity imaging by coupling a simple BODIPY rotor with a mitochondria-targeting unit. The **BTV** exhibited a significant fluorescence intensity enhancement of more than 100-fold as the solvent viscosity increased. Also, the probe showed a direct linear relationship between the fluorescence lifetime and the media viscosity, which makes it possible to trace the change of the medium viscosity. Furthermore, it was demonstrated that **BTV** could achieve practical applicability in the monitoring of mitochondrial viscosity changes in live cells through fluorescence lifetime imaging microscopy (FLIM).

## 1. Introduction

Intracellular viscosity has an important influence on transportation as well as the interaction of biomolecules and chemical signals between biomacromolecules [1,2,3]. Also, it has influences on diffusion in biological processes, such as cell apoptosis and the transportation of small solutes and other cellular organelles in living cells [4]. It has been demonstrated that the abnormal changes of intracellular viscosity are related to a large variety of diseases and pathologies, such as Alzheimer’s disease [5], atherosclerosis [6], and diabetes [7]. In view of the important role of cellular viscosity in cell functioning and pathogenic conditions, the development of a cellular viscosity sensor for understanding intracellular reaction kinetics and the development of biological research and clinical diagnosis strategies has attracted a great deal of attention [8,9]. Fluorescence imaging is a powerful technique to study biological systems in living cells due to their high sensitivity and easy visibility. To map the cellular viscosity, a group of molecular rotors, by using fluorescence emission intensity or fluorescence lifetime imaging, were developed as microviscosity sensors [4,10,11,12]. Most fluorescent molecular rotors (FMRs) based on a twisted intramolecular charge transfer (TICT) mechanism, in which the non-radiative decay of an excited state can be altered by the surrounding viscosity, have been developed for their rapid response and high spatial resolution [1,13,14,15,16,17,18,19,20,21]. Further, Förster resonance energy transfer (FRET) was introduced to intracellular viscosity sensor development to remove the influence of concentration-related artifacts [22,23]. Up to now, these designs have often involved a limited number of FMRs or multistep synthetic protocols, which makes them inconvenient to acquire or inappropriate for further improvement. Furthermore, most of these probes showed non-specific intracellular distribution, which makes them difficult to use for the mapping of the change of viscosities in specific organelles. In order to monitor the change of viscosities of a specific organelle with high sensitivity, new methods with simple and efficient ways to design fluorescent viscosity probes are required.

Mitochondria are essential for the maintenance of cell function, and are often described as the power house of the cell [24]. The viscosity of mitochondria reflects the status and function of the organelle, and its function can be influenced by changes in the mitochondrial membrane viscosity, the potentialization of reactive oxygen species (ROS) production and cytochrome c release [24,25]. With the importance of membrane viscosity in mitochondrial dysfunction in mind, the development of sensitive and specific probes towards sensing viscosity in mitochondria is of critical importance for the understanding of its role in diseases [26,27]. The measurement of viscosity changes in mitochondria can be achieved by affixing an organelle-specific group to efficient fluorescent molecular rotors [8,23,28].

Rotor efficiency has become a stumbling block in the development of viscosity sensors. There is an urgent need to develop a new fluorescence rotor with simple synthetic protocols, low background and high efficiency. Boron-dipyrromethene (BODIPY) dyes have been extensively used as fluorophores, due to their high molar absorption coefficients, sharp fluorescence peaks with high quantum yields and because they are relatively insensitive to the polarity and pH of the environment [29]. In our previous work, we reported a novel dark BODIPY structure, where the intramolecular rotation of the phenyl ring around the BODIPY core led to near non-fluorescence [30,31]. The experimental data showed that by increasing the viscosity of the solvent, the fluorescence intensity of this dark BODIPY dye was dramatically enhanced (more than 100-fold) and the quantum yield changed from 0.01 to 0.57 [30]. Inspired by the features of this dark BODIPY dye, we aimed to develop a simple BODIPY-based viscosity probe for mitochondria with low background and high sensitivity.

**Scheme 1:**
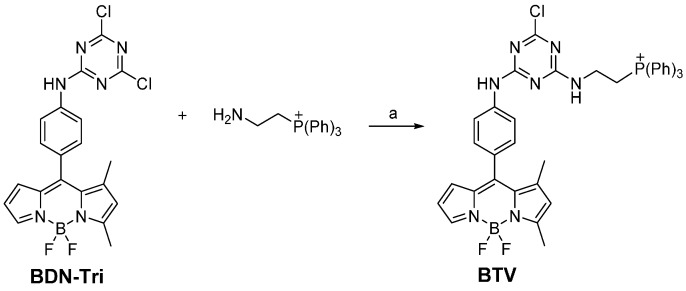
General synthetic scheme of **BTV**. Reagents and conditions: a: THF, DIEA, 2 h.

On the basis of these studies, we developed **BTV**, a mitochondria molecular viscosity probe, by attaching a triphenylphosphonium moiety as a mitochondria-targeting unit to a simple BODIPY dye with only one side of methyl-substituted pyrrole functioning as the rotor (Scheme 1). Based on our previous knowledge, the fluorescent reporter is likely to show high sensitivity towards viscosity due to the intramolecular rotation of the phenyl ring. The free rotating of the rotor results in the increased non-radiative decay rate and consequently reduced fluoscence quenching in a low viscosity environment, whereas restricted rotation produces fluorescence enhancement in a high viscosity environment.

## 2. Materials and Methods 

### 2.1. In Vitro Fluorescence Lifetime Detection

Fluorescence lifetime was measured by using time-correlated single photon counting (TCSPC) technique based on PicoHarp 300 module. A mode-locked Ti:sapphire oscillator (Avesta Ti-Saphire TiF-100M) was utilized as excitation source, which outputting 80 fs laser pulses with a central wavelength at 860 nm and a repetition rate of 84.5 MHz. The laser was frequency doubled to 430 nm by using a BBO crystal and filtered by a 750 nm short pass before focusing on the sample. The emission signal was sent to a monochromator (Acton, Spectra Pro 2300 i) centered at 510 ± 5 nm and detected by a photon counting photomultiplier detector (PicoQuant, PMA 182-N-M) to obtain the fluorescence decay curve. The instrument response function (IRF) was measured by using Ludox solution. The lifetime was obtained by deconvolving the IRF from the fluorescence decay curve.

### 2.2. Cell Incubation and Imaging

HeLa cells were cultured in high-glucose (4500 mg/L) containing-Dulbecco’s Modified eagle’s medium (DMEM) supplemented with 10% fetal bovine serum, 100 U/mL penicillin and 100 µg/mL streptomycin. Then 24–36 h prior to imaging, cells was seeded in clear bottom, 96-well plate. **BTV** was added to cultured cells to reach final concentration of 1 µM and incubated for 30 min at 37 °C and subsequently washed twice with PBS buffer before imaging. For mitochondria co-localization study, 0.5 µM MitoTracker Deep Red (Life Technologies, Waltham, MA, USA) was incubated together with **BTV**. To induce mitochondria swelling, cells were pre-treated with 10 µM monensin for 30 min at 37 °C before **BTV** staining. Live cell images were acquired on an inverted Ti-E microscope (Nikon Instruments Inc., Tokyo, Japan), equipped with FITC filter for **BTV** fluorescence acquisition, as well as Cy5 for MitoTracker DeepRed fluorescence acquisition. Images were analysed using NIS Elements 3.10 software.

The time domain FLIM experiments were performed on Time-Correlated Single Photon Counting (TCSPC) system (Picoquant, Germany) attached with Olympus FV-1000 inverted confocal microscope (Olympus, Singapore) with a 60 × 1.2 W objective. The excitation light source was 485 nm pulsed diode laser controlled by a Sepia II (PioQuant) driver having a dichroic mirror of 488/559 and 520/30 emission filter. Individual photon arrivals were detected using a SPAD detector and events were recorded by a PicoHarp 300 TCSPC module. Lifetime analysis was carried out using Symphotime (PicoQuant) software, mono exponential fittings were performed to observe viscosity induced lifetime changes.

### 2.3. Synthesis of **BTV**

Precursor **BDN-Tri** was synthesized as reported [30,31]. **BDN-Tri** (30 mg, 65 µmol), (2-aminoethyl)triphenylphosphonium (20 mg, 65 µmol) and DIEA (17 µL,130 µmol) were dissolved in THF (4 mL) and stirred at room temperature for 2 h. After evaporation of the solvent, the crude product was purified by silica gel chromatography (eluents: CH_2_Cl_2_ to CH_2_Cl_2_: MeOH (95:5)) to afford the corresponding **BTV** as orange solid (29.4 mg, 62%). ^1^H NMR (500 MHz, CDCl_3_): *δ* = 7.92–7.75 (m, 10H), 7.71–7.63 (m, 9H), 7.24 (s, 1H), 6.40 (s, 1H), 6.34 (s, 1H), 6.11 (s, 1H), 4.04–3.95 (m, 4H), 2.60 (s, 2H), 1.58 (s, 3H), 1.25 (s, 3H). ^13^C NMR (125 MHz, CDCl_3_): 167.59, 161.78, 160.28, 146.91, 142.99, 135.15, 135.08, 133.84, 133.76, 133.64, 133.56, 130.50, 130.40, 129.52, 129.36, 126.92, 123.18, 121.03, 118.08, 117.39, 115.90, 29.57, 15.33, 15.06, 14.01. HRMS *m/z* (C_40_H_35_BClF_2_N_7_P^+^) calculated: 728.2436, found: 728.2464.

## 3. Results

### 3.1. Fluorescence Response to Solvent Viscosity

**Figure 1:**
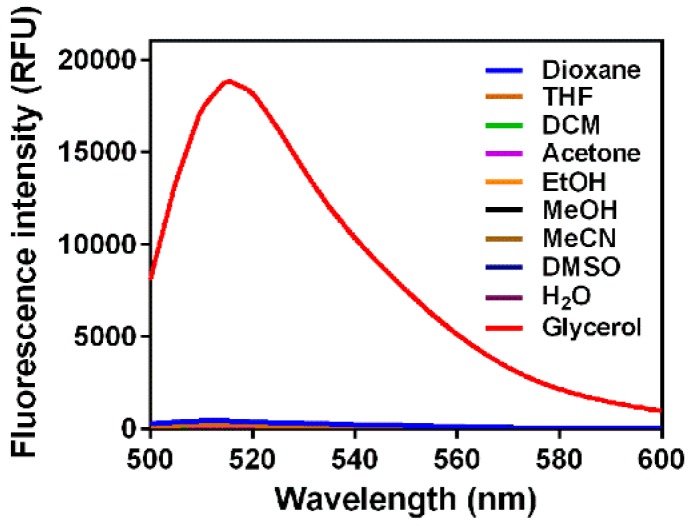
Fluorescence intensity of **BTV** (10 µM, *λ*_ex_ = 470 nm, 25 °C) in different solvents.

The solvent dependency of **BTV** has been studied by measuring the spectroscopic properties of **BTV** in various solvents with distinct viscosity properties. The maximum absorption and emission bands of **BTV** did not display significant changes. In contrast, the fluorescence intensity of **BTV** in glycerol was obviously larger than that in other organic solvents. The quantum yields of **BTV** in different solvents have been summarized in Table S1, which showed that the quantum yields were less than 0.1 in low-viscosity media, whereas in high-viscosity solvent, such as glycerol, the quantum yield increased to 0.56 (Figure 1). Based on our previous observation, the increased quantum yield of **BTV** in high-viscosity solvent was a result of the restricted intramolecular rotation of the phenyl rings around the axes of the single bond linked to the BODIPY core [14,30]. In the presence of highly viscous solvent, the absorbed energy was dissipated predominantly through the radiative emission channel rather than lost through rotational energy relaxation. On the other hand, the relationship between the solvent polarity and the fluorescence intensity of **BTV** was also investigated. It was found that the fluorescence intensity was not affected by the solvent polarity, which makes it beyond the influence of environmental conditions (Table S1).

**Figure 2:**
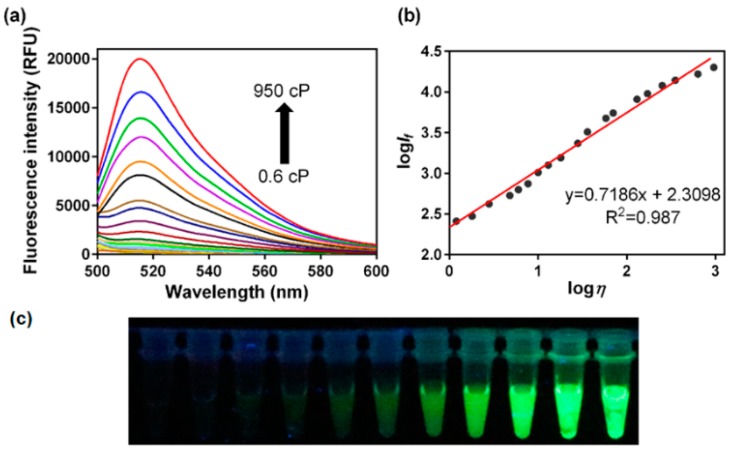
Viscosity-dependent fluorescence spectra of **BTV** in solvent mixtures. (**a**) Changes of fluorescence spectra of **BTV** (10 μM, *λ*_ex_ = 470 nm, 25 °C) with the variation of solution viscosity (methanol-glycerol system); (**b**) The linear response between log *I*_515_ and log *η*; (**c**) Color and fluorescence changes of **BTV** (10 μM) in mixed solvents (methanol-glycerol) with various viscosities under a UV lamp (365 nm excitation).

The relationship between viscosity (*η*) and fluorescence intensity (*I_f_*) was investigated. With the increase of the viscosity, it was expected that the rotation of the phenyl rings will be hampered and the fluorescence of **BTV** will be restored. As shown in Figure 2, we investigated the emission spectra of **BTV** in a system consisting of mixtures of methanol and glycerol. The viscosities of the solutions were increased from 0.6 cP (100% methanol) to 950 cP (100% glycerol) by increasing the portion of glycerol in the methanol [32]. From the experimental data, it is clear that the fluorescence intensity of **BTV** was greatly enhanced at 515 nm by more than 100-fold and the quantum yield changed from 0.01 to 0.56 (Figure 2a)**.** To our knowledge, **BTV** shows the best fluorescence intensity fold increase compared to other reported molecular rotors for solvent viscosity in the range of 0.6 cP (methanol) to 950 cP (glycerol) (Table S2), which indicates that **BTV** exhibits considerably higher sensitivity for solvent viscosity [8,16,33,34]. The quantitative relationship between the fluorescence intensity *I_515_* of **BTV** and the solvent viscosity *η* was created according to the Förster-Hoffmann equations:

(1) $$\log I_{f}=C+\chi \log\eta$$

where *I_f_* is the fluorescent emission intensity, *η* is the solvent viscosity, *C* is a concentration- and temperature-dependent constant, and χ is the dye-dependent constant, which shows the sensitivity of the probe towards viscosity [35]. The results show that the plot of the **BTV** fits the linear relationship between log *I*_515_ and log *η* (R^2^ = 0.987, χ = 0.718). Taken together, the **BTV** could be used for the detection of the viscosity changes in various media.

### 3.2. Fluorescence Lifetime Response to Solvent Viscosity 

**Figure 3:**
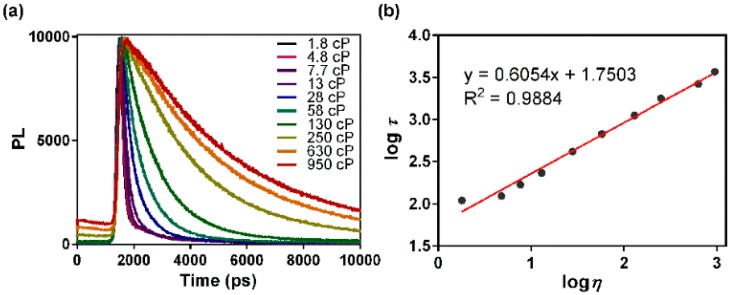
Viscosity-dependent fluorescence lifetime of **BTV** in solvent mixtures. (**a**) Changes of fluorescence lifetime of **BTV** (10 μM, *λ*_ex_ = 430 nm, 25 °C) with the variation of solution viscosity (methanol-glycerol system); (**b**) The linear response between log *τ* and log *η*.

In addition, the fluorescence lifetime measurement of the **BTV** was also performed to study the relationship between the viscosity (*η*) and fluorescence lifetime (*τ_f_*) of the **BTV**. Determination of viscosity by fluorescence intensity measurement suffers from the changes of medium properties and variations in dye concentrations, which can be more severe in live cell conditions [22]. The fluorescence lifetime, on the other hand, is independent of the fluorescence intensity and fluorophore concentration which allows us to quantify the viscosity in live cells [33,36]. As shown in Figure 3a, the lifetime (*τ_f_*) of the **BTV** at 515 nm was gradually increased from 0.1 ns to 3.7 ns with increasing the solution viscosity from 1.8 cP to 950 cP. The observed increase in the fluorescence lifetime is consistent with the restricted rotation of the phenyl group in the medium of high viscosity, thus preventing relaxation via non-radiative decay. The quantitative relationship between the fluorescence lifetime (*τ_f_*) of the **BTV** and the solvent viscosity (*η*) was created according to the Förster-Hoffmann equation:

(2) $$\log\tau_{f}=C+\chi \log\eta$$

where *τ_f_* is the fluorescence lifetime, *η* is the solvent viscosity, *C* is a concentration- and temperature-dependent constant, and χ is the dye-dependent constant [35]. A linear relationship was observed between the lifetime (*τ_f_*) and solvent viscosity (*η*) (R^2^ = 0.988, χ = 0.605) (Figure 3b and Table S3) [33]. Hence, the viscosity-lifetime curve could be used as a calibration curve for the quantification of the viscosity by fluorescence lifetime imaging microscopy (FLIM) [36].

### 3.3. Application of **BTV** in Live Cells 

**Figure 4:**
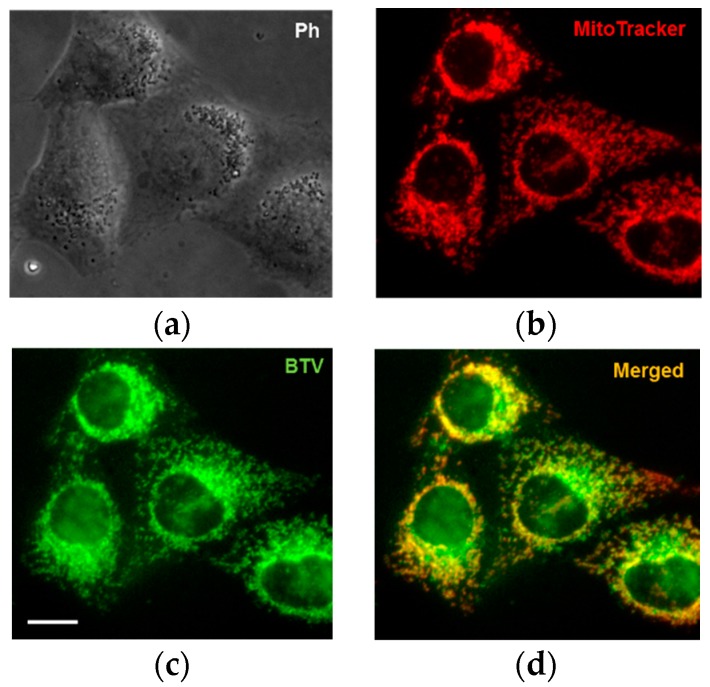
Fluorescence microscopic images of **BTV** (1 μM) in HeLa cells with 0.5 μM MitoTracker. DeepRed. Images were taken with a 40× objective. Scale bar represents 20 μm. (**a**) Phase contrast; (**b**) MitoTracker DeepRed; (**c**) **BTV**; (**d**) Merged of MitoTracker DeepRed and **BTV**.

Since in vitro spectroscopic data of the **BTV** had displayed both fluorescence intensity and lifetime sensitivity towards the viscosity of the environment, we then examined the potential applications of the **BTV** in fluorescent live cell imaging. For measuring the viscosity of cell mitochondria using the **BTV**, the intracellular location of the dye was first established. HeLa cells were incubated with **BTV** and MitoTracker DeepRed for 30 min at 37 °C before fluorescence microscopic images were taken. The merged image from the bright-green fluorescence of the **BTV** and red fluorescence of the MitoTracker showed good co-localization (Pearson’s correlation = 0.87). This confirms that the **BTV** mainly localized in the mitochondria because of its triphenylphosphonium moiety, with marginal staining in the cell membrane (Figure 4). The staining of the cell membrane by **BTV** is likely to be due to the highly liposoluble BODIPY structure in the **BTV** [19].

**Figure 5:**
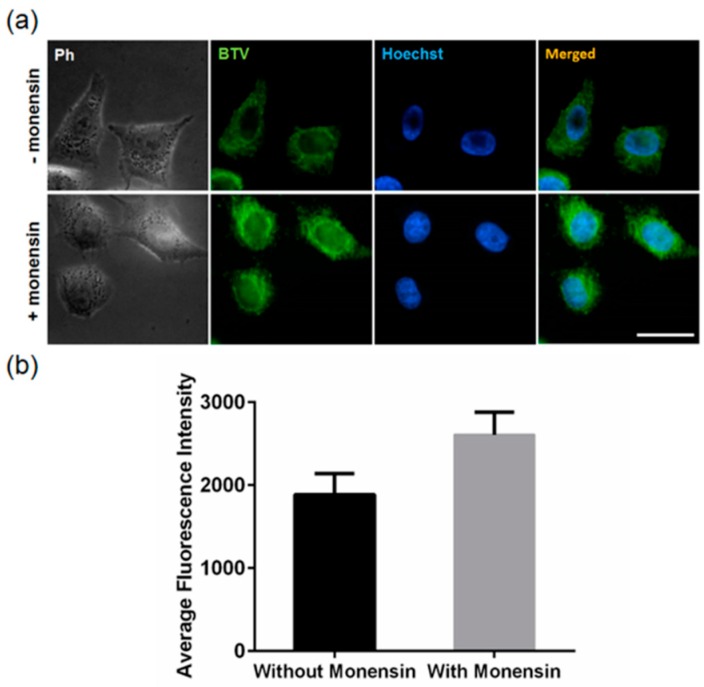
(**a**) Fluorescence intensity measurement of **BTV**-treated (1 μM) HeLa cells before and after incubation with 10 μM monensin for 30 min. Images were taken with a 40× objective. Scale bar represents 50 μm; (**b**) Fluorescence intensities were displayed as averages of measurements from twelve sites taken across three wells. * *p* < 0.005.

To demonstrate the capability of the **BTV** to monitor viscosity changes in mitochondria, fluorescence imaging of HeLa cells was acquired in the presence of an ionophore, monensin, which can induce mitochondrial malfunction caused by structural changes or vacuolization and/or swelling of the mitochondria because of the interruption of the ionic balance [23,37,38]. HeLa cells were pre-stimulated by a low dose of monensin, in order to alter the mitochondrial viscosity. Compared to untreated cells, monensin-treated cells kept their shape but the average fluorescence intensity of cell imaging had increased (Figure 5). From the linear relationship between the fluorescence intensity and viscosity established in Figure 2b, an increase in the average viscosity of mitochondria in monensin-treated HeLa cells was observed. At the same time, the results also agree with previous findings that this ionophore can induce ultrastructural changes or swelling of the mitochondria in its metabolism [38]. Taken together, these results provide sufficient evidence for the validity of **BTV** as a viscosity sensor for viscosity changes in mitochondria.

**Figure 6:**
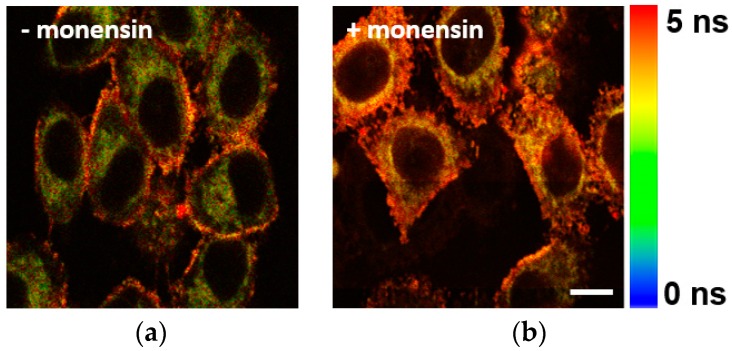
Fluorescence lifetime images of **BTV**-treated (1 μM) HeLa cells (**a**) Before and (**b**) After incubation with 10 μM monensin for 30 min. Scale bare represents 20 μm.

To quantify the specific viscosity change in mitochondria using **BTV**, the fluorescence lifetime imaging (FLIM) experiment was conducted (Figure 6). It can be seen that the cell mitochondria are colored a bluish-green, indicating the fluorescence lifetime of the **BTV** in mitochondria to be less than 2.5 ns from the color bar. Upon treatment with monensin, the fluorescence lifetime of the **BTV** in mitochondria was enhanced from 2.77 ns to 3.17 ns (*p* < 0.05), as reflected by the color change to a greenish-yellow, which shows an increase in the viscosity after the treatment of monensin. On the other hand, we can see the **BTV**-stained cell membrane, outlined in orange color, with no significant change in its measured lifetime from 3.54 ns to 3.63 ns (*p* > 0.05) upon monensin treatment. This confirms that **BTV** responded only to viscosity changes in the mitochondria due to monensin treatment, and hence can be used for the monitoring of mitochondrial viscosity changes in live cells.

## 4. Conclusions 

In conclusion, we have developed a mitochondrial viscosity probe, **BTV**, by connecting a simple BODIPY rotor with a mitochondria-targeting unit. **BTV** shows weak emissions due to the fast intramolecular rotation between the phenyl rings and the BODIPY core. The **BTV** fluorescence intensity showed an enhancement of more than 100-fold with increased solvent viscosity, while the fluorescence lifetimes of the **BTV** displayed a good linear relationship with the viscosity. All the characteristics enabled the **BTV** to be applied for the monitoring and imaging of mitochondrial viscosity changes by FLIM. Also, the **BTV** has illustrated a significant increase in mitochondrial viscosity by treatment with monensin. In this report, we have presented an optional tool that may help in the diagnosis of certain mitochondria-related diseases.

## Supplementary Materials

The following are available online at http://www.mdpi.com/1424-8220/16/9/1397/s1, Experimental Procedures, Table S1. Spectral data of **BTV** in different solvents. Table S2. Examples of viscosity sensors. Table S3. Viscosity-dependent fluorescent lifetime of **BTV** in solvent mixtures.
