# Supplementary material for: A Simple BODIPY-Based Viscosity Probe for Imaging of Cellular Viscosity in Live Cells
Source: Sensors (Basel). 2016 Aug 31;16(9):1397. doi: 10.3390/s16091397 (PMC5038675; doi:10.3390/s16091397)
Supplement: Supplementary file 1 [file sensors-16-01397-s001.pdf]

Supplementary Materials: A Simple BODIPY-Based Viscosity Probe for Imaging of Cellular Viscosity in Live Cells

Dongdong Su, Chai Lean Teoh, Nengyue Gao, Qing-Hua Xu and Young-Tae Chang

1. Experimental Procedures

1.1. Material and Method

All reactions were performed in oven-dried glassware under a positive pressure of nitrogen. Unless otherwise noted, starting materials and solvents were purchased from Aldrich and Acros organics and used without further purification. NMR spectra were recorded on a Bruker AMX500 (500 MHz) NMR spectrometer. Chemical shifts are reported as δ in units of parts per million (ppm) and coupling constants are reported as a *J* value in Hertz (Hz). Spectroscopic and quantum yield data were measured on a SpectraMax M2 spectrophotometer (Molecular Devices), compounds are dissolved in 100 μL solvent in 96-well plates for λ_{em}. Data analysis was performed using Graph Prism 5.0.

1.2. Quantum Yield Measurements

Quantum yields for fluorescent compounds were measured by dividing the integrated emission area of their fluorescent spectrum against the area of rhodamine B in EtOH excited at 490 nm ($\Phi_{rho\text{-}B}$ = 0.7). Quantum yields where then calculated using Equation (1), where *F* represents the integrated emission area of fluorescent spectrum, η represents the refractive index of the solvent, and *Abs* represents absorbance at excitation wavelength selected for standards and samples. Emission was integrated from 500 nm to 700 nm.

$$\Phi_{flu}^{sample} = \Phi_{flu}^{reference}\left(\frac{F^{sample}}{F^{reference}}\right)\left(\frac{\eta^{sample}}{\eta^{reference}}\right)\left(\frac{Abs^{reference}}{Abs^{sample}}\right) \quad (1)$$

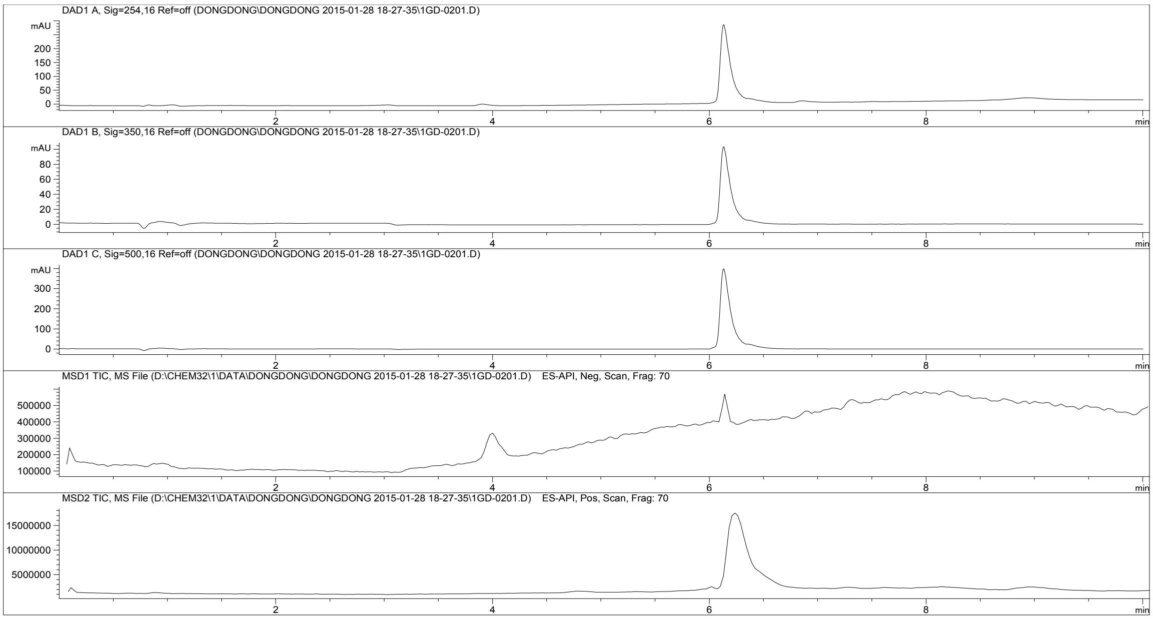

(**a**)

Figure S1. *Cont.*

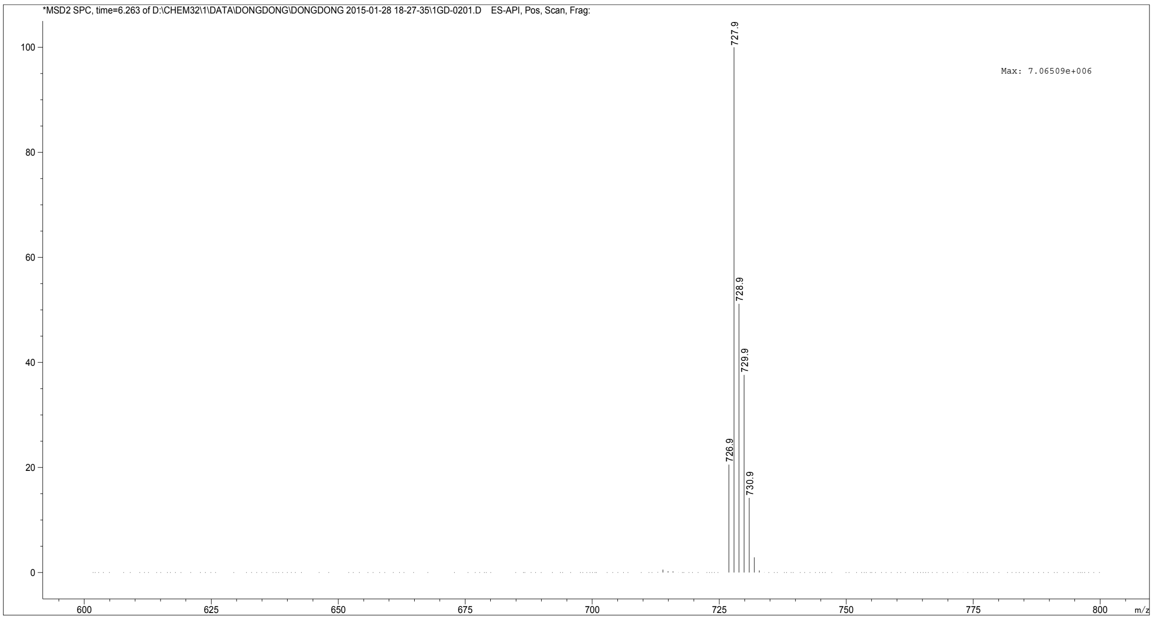

(**b**)

Figure S1. LC-MS data of **BTV** (**a**) Chromatograms (descending order) at 254 nm, 350 nm and 500 nm; (**b**) ESI-MS positive spectra. HPLC-MS (Agilent-1200 series) with a DAD detector and a single quadrupole mass spectrometer (6130 series) with an ESI probe. Analytical HPLC method: eluents, A: H_2O (0.1% HCOOH), B: CH_3CN (0.1% HCOOH), gradient 5% B to 95% B (10 min). Reverse-phase Phenomenex C18 Luna column (4.6x50mm^2, 3.5mm particle size), flow rate: 1 mL/min.

Table S1. Spectral data of **BTV** in different solvents.

	**Dielectric Constant**	η **(cP)** [a]	λ_{abs} **(nm)**	λ_{em} **(nm)**	Φ [b]
Dioxane	2.2	1.54	494	510	0.02
THF	7.6	0.53	494	514	0.02
DCM	9.1	0.43	494	514	0.02
Acetone	20.7	0.32	490	510	0.01
EtOH	24.3	1.20	490	510	0.02
MeOH	32.6	0.60	490	510	0.01
MeCN	37.5	0.37	490	510	0.01
DMSO	48.9	2.24	494	515	0.03
H_2O	78.4	1.01	490	514	0.01
Glycerol	45.8	950.17	494	515	0.56

[a] Viscosity of the solvents. [b] Fluorescence quantum yields were measured using rhodamine B (Φ = 0.7 in EtOH) as a standard.

Table S2. Examples of viscosity sensors.

Name	Sensor Structure	Viscosity Change	Fluorescence Fold Change	Reference
RY3	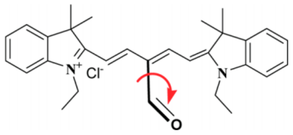	1.2 cP (ethanol) to 950 cP (99% glycerol)	6 times	[1] *J. Am. Chem. Soc.* **2011**, *133*, 6626–6635
meso-substituted 4,4′-difluoro-4-bora-3a, 4adiaza-*s*-indacene (**1**)	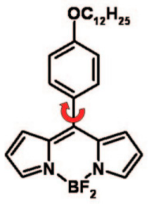	0.6 (Methanol) to 950 cP (99% glycerol)	12 times	[2] *J. Am. Chem. Soc.* **2008**, *130*, 6672–6673
Bodipy 2	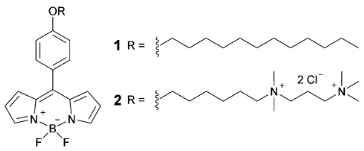	0.5 to 950 cP	39 times	[3] *Chem. Commun.* **2014**, *50*, 5282–5284
	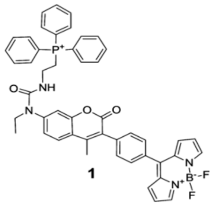	0.6 (Methanol) to 950 cP (99% glycerol)	15 times	[4] *J. Am. Chem. Soc.* **2013**, *135*, 9181–9185

Table S2. *Cont.*

Name	Sensor Structure	Viscosity Change	Fluorescence Fold Change	Reference
	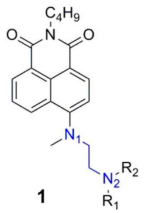	20.5 to 945 cP.	34 times	[5] *Sci. Rep.* **2014**, *4*, 5418.
Caz-Cy2	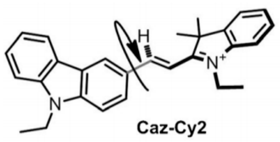	1.01 (water) to 950 (glycerol)	80 times	[6] *Chem. Eur. J.* **2013**, *19*, 1548–1553
BV1	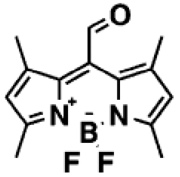	1.01 (water) to 950 (glycerol)	50 times	[7] *Chem. Eur. J.* **2014**, *20*, 4691–4696
Rotor 1	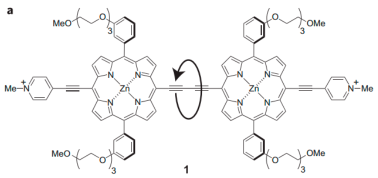	0.6 (Methanol) to 950 cP (99% glycerol)	12times	[8] *Nat. Chem.* **2009**, *1*, 69
Mito-V	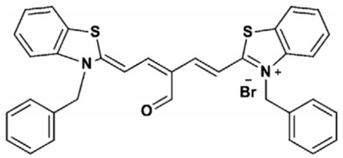	1.1 (water) to 871 cP (90% glycerol)	63 times	[9] *Sensors and Actuators B* **2014**, *19*, 685–693

Table S3. Viscosity-dependent fluorescence lifetime of **BTV** in solvent mixtures.

Portion of Glycerol	η (cP)	τ (ps)	log η	log τ
0.1	1.8	109	0.255273	2.037426
0.2	4.8	124	0.681241	2.093422
0.3	7.7	168	0.886491	2.225309
0.4	13	232	1.113943	2.365488
0.5	28	416	1.447158	2.619093
0.6	58	672	1.763428	2.827369
0.7	130	1113	2.113943	3.046456
0.8	250	1775	2.39794	3.249076
0.9	630	2650	2.799341	3.423246
0.99	950	3690	2.977724	3.566991

References

1. Peng, X.; Yang, Z.; Wang, J.; Fan, J.; He, Y.; Song, F.; Wang, B.; Sun, S.; Qu, J.; Qi, J.; et al. Fluorescence ratiometry and fluorescence lifetime imaging: Using a single molecular sensor for dual mode imaging of cellular viscosity. *J. Am. Chem. Soc.* **2011**, *133*, 6626–6635.
2. Kuimova, M.K.; Yahioglu, G.; Levitt, J.A.; Suhling, K. Molecular rotor measures viscosity of live cells via fluorescence lifetime imaging. *J. Am. Chem. Soc.* **2008**, *130*, 6672–6673.
3. Lopez-Duarte, I.; Vu, T.T.; Izquierdo, M.A.; Bull, J.A.; Kuimova, M.K. A molecular rotor for measuring viscosity in plasma membranes of live cells. *Chem. Commun.* **2014**, *50*, 5282–5284.
4. Yang, Z.; He, Y.; Lee, J.H.; Park, N.; Suh, M.; Chae, W.S.; Cao, J.; Peng, X.; Jung, H.; Kang, C.; Kim, J.S. A self-calibrating bipartite viscosity sensor for mitochondria. *J. Am. Chem. Soc.* **2013**, *135*, 9181–9185.
5. Liu, T.; Liu, X.; Spring, D.R.; Qian, X.; Cui, J.; Xu, Z. Quantitatively mapping cellular viscosity with detailed organelle information via a designed PET fluorescent probe. *Sci. Rep.* **2014**, *4*, 5418.
6. Liu, F.; Wu, T.; Cao, J.F.; Cui, S.; Yang, Z.G.; Qiang, X.X.; Sun, S.G.; Song, F.L.; Fan, J.L.; Wang, J.Y.; Peng, X.J. Ratiometric Detection of Viscosity Using a Two-Photon Fluorescent Sensor. *Chem-Eur. J.* **2013**, *19*, 1548–1553.
7. Zhu, H.; Fan, J.L.; Li, M.; Cao, J.F.; Wang, J.Y.; Peng, X.J. A "Distorted-BODIPY"-Based Fluorescent Probe for Imaging of Cellular Viscosity in Live Cells. *Chem-Eur. J.* **2014**, *20*, 4691–4696.
8. Kuimova, M.K.; Botchway, S.W.; Parker, A.W.; Balaz, M.; Collins, H.A.; Anderson, H.L.; Suhling, K.; Ogilby, P.R. Imaging intracellular viscosity of a single cell during photoinduced cell death. *Nat. Chem.* **2009**, *1*, 69–73.
9. Jiang, N.; Fan, J.L.; Zhang, S.; Wu, T.; Wang, J.Y.; Gao, P.; Qu, J.L.; Zhou, F.; Peng, X.J. Dual mode monitoring probe for mitochondrial viscosity in single cell. *Sens. Actua. B Chem.* **2014**, *190*, 685–693.
